# Neurovascular imaging with QUTE-CE MRI in APOE4 rats reveals early vascular abnormalities

**DOI:** 10.1371/journal.pone.0256749

**Published:** 2021-08-27

**Authors:** Joshua Leaston, Craig F. Ferris, Praveen Kulkarni, Dharshan Chandramohan, Anne L. van de Ven, Ju Qiao, Liam Timms, Jorge Sepulcre, Georges El Fakhri, Chao Ma, Marc D. Normandin, Codi Gharagouzloo

**Affiliations:** 1 Imaginostics, Inc., Cambridge, Massachusetts, United States of America; 2 Department of Psychology, Northeastern University, Boston, Massachusetts, United States of America; 3 Center for Translational Neuroimaging, Northeastern University, Boston, Massachusetts, United States of America; 4 Department of Physics, Northeastern University, Boston, Massachusetts, United States of America; 5 Nanomedicine Science and Technology Center, Northeastern University, Boston, Massachusetts, United States of America; 6 Gordon Center for Medical Imaging, Massachusetts General Hospital and Harvard Medical School, Boston, Massachusetts, United States of America; Henry Ford Health System, UNITED STATES

## Abstract

Cerebrovascular abnormality is linked to Alzheimer’s disease and related dementias (ADRDs). ApoE-Ɛ4 (APOE4) is known to play a critical role in neurovascular dysfunction, however current medical imaging technologies are limited in quantification. This cross-sectional study tested the feasibility of a recently established imaging modality, quantitative ultra-short time-to-echo contrast-enhanced magnetic resonance imaging (QUTE-CE MRI), to identify small vessel abnormality early in development of human APOE4 knock-in female rat (TGRA8960) animal model. At 8 months, 48.3% of the brain volume was found to have significant signal increase (75/173 anatomically segmented regions; q<0.05 for multiple comparisons). Notably, vascular abnormality was detected in the tri-synaptic circuit, cerebellum, and amygdala, all of which are known to functionally decline throughout AD pathology and have implications in learning and memory. The detected abnormality quantified with QUTE-CE MRI is likely a result of hyper-vascularization, but may also be partly, or wholly, due to contributions from blood-brain-barrier leakage. Further exploration with histological validation is warranted to verify the pathological cause. Regardless, these results indicate that QUTE-CE MRI can detect neurovascular dysfunction with high sensitivity with APOE4 and may be helpful to provide new insights into health and disease.

## 1. Introduction

Age-related dementia affects over 50 million people worldwide. In the US, Alzheimer’s disease (AD) affects nearly six million people, with annual costs for long-term care and hospice-services close to $300BN [1]. Aging also increases the risk of cerebrovascular disease (CVD), long considered to be a leading cause of dementia [2,3]. Accumulating evidence indicates that CVD also plays a key role in neurodegeneration and in AD and related dementia (ADRD) [4,5]. In addition, close to 26% of dementia is considered to be primarily vascular in origin, and vascular impairment is associated with all major dementias. Dementia presents most commonly as amyloid changes in vessel walls. These changes include narrowed vessels, hypoperfusion, and capillary vasoconstriction [6]. Blood-Brain-Barrier (BBB) leakage is also associated with aging in disease pathology [7]. Recently, results from the AD Neuroimaging Initiative (ADNI) identified vascular dysregulation as the earliest and most significant biomarker for late onset AD (LOAD)—even before amyloid beta accumulation [8]. Though literature is divided on the subject, there is a minority opinion that a paradigm shift is presently underway in the research community, with a new emphasis on the association of vascular pathophysiology and AD [9].

While over 21 genes have now been associated with LOAD, ApoE is associated with the highest degree of confidence [10]. ApoE are primarily produced by astroglia and microglia to transport lipids to neurons, which have an abundance of surface receptors in the CNS [11,12]. ApoE exists in three isoforms (E2, E3 and E4), which differ from each other by one or two amino acid substitutions at residues 112 (E2) and 158 (E4 [12]). Having two copies of ApoE4 (approximately 2% of the population) puts someone at a 60% risk to develop AD by age 85, whereas a 25% likelihood to develop AD by 85 exists with just one copy. Studies indicate that abnormality appears decades in advance of disease onset [13–15] for humans that carry the ApoE-Ɛ4 gene.

Conventional magnetic resonance imaging (MRI) of vascular abnormality includes a variety of qualitative modalities, including T2/fluid-attenuated inversion recovery, for identifying ischemic lesions as white matter hyperintensities; diffusion-weighted imaging, for identifying acute infarcts; and susceptibility-weighted imaging, to identify cerebral microbleeds [16]. Dynamic susceptibility contrast MRI is the gold standard for quantitative cerebral blood volume (CBV) imaging, but is limited by inaccurate contrast agent concentration measurements [17,18], with signal dependent on vessel orientation, susceptibility, and baseline drift [19] making it inaccurate for precision medicine. Functional MRI (fMRI) uses the BOLD signal to describe network connectivity [19] and has been used to measure vascular-related metabolic compensation occurring in both young and old ApoE-Ɛ4 carriers [13–15]. Arterial spin labeling (ASL)–which isolates the CBF [20,21]–has been used to identify patterns of hypo- or hyper-perfusion that correlate both to age and cognitive decline in ApoE-Ɛ4 carriers [22,23]. In addition to CBF-related perfusion, dynamic contrast-enhanced (DCE) MRI performed with gadolinium-based contrast agents (GBCAs), has been used to measure CBV changes in the hippocampus that correlate to cognitive impairment in the early to late stages of AD [24]. These techniques allow clinicians to detect the gross neuropathological consequences of small vessel disease, but they do not address the function or state of the microvasculature itself, and thus fall short of assessing the quantitative burden of the disease state [25,26].

In this study, comparative analyses were conducted between age-matched female ApoE-Ɛ4 (APOE4) rats and Sprague Dawley wild-type (WT) littermate controls. Quantitative ultra-short time-to-echo contrast-enhanced (QUTE-CE) is a recently established quantitative CBV-based vascular imaging modality [27–31] which has been used to analyze the small vessel density in healthy rats [28], and BBB leakage in a diabetic model [31]. Here, we investigate the relationship between the APOE4 phenotype and vascular abnormality early in development (8 months of age; approximately 22-year-old human age [32]). Unlike other MRI-based measurements [19], the use of a 3D non-slice selective pulse and an ultra-short echo time removes signal modulation due to magnetic susceptibility, vessel orientation and blood flow to produce a quantitative signal intensity [27]. Selective enhancement of the vascular compartment with an intravascular contrast agent allows quantitative measurements of vascular physiology [28,30].

## 2. Methods

### 2.1 Experimental design

Wild-type (WT) and human ApoE-Ɛ4 knock-in (TGRA8960) female Sprague Dawley rats were obtained from Horizon Discovery (St. Louis, MO). Rats were housed in pairs in Plexiglas cages, in ambient temperature (22–24°C), on a 12h:12h light-dark cycle (lights on at 7:00 am). Food and water were provided *ad libitum*. Rats were imaged during the light phase of the circadian cycle. All rats were acquired and cared for in accordance with the guidelines published in the National Institute of Health (NIH) Guide for the Care and Use of Laboratory Animals. All methods and procedures described below were pre-approved by the Northeastern University Institutional Animal Care and Use Committee. The protocols used in this study adhere to the ARRIVE guidelines for reporting *in vivo* experiments in animal research [33].

This cross-sectional study was performed on a subset of female rats. Images were obtained at 8 months of age. Two groups (n = 5 WT and n = 6 APOE4) were imaged. Animals were effectively the same size with no significant difference in weight or brain sizes (S1 Fig). After this study, the rats were returned to their housing for use in other studies.

### 2.2 Magnetic resonance imaging

**Fig 1:**
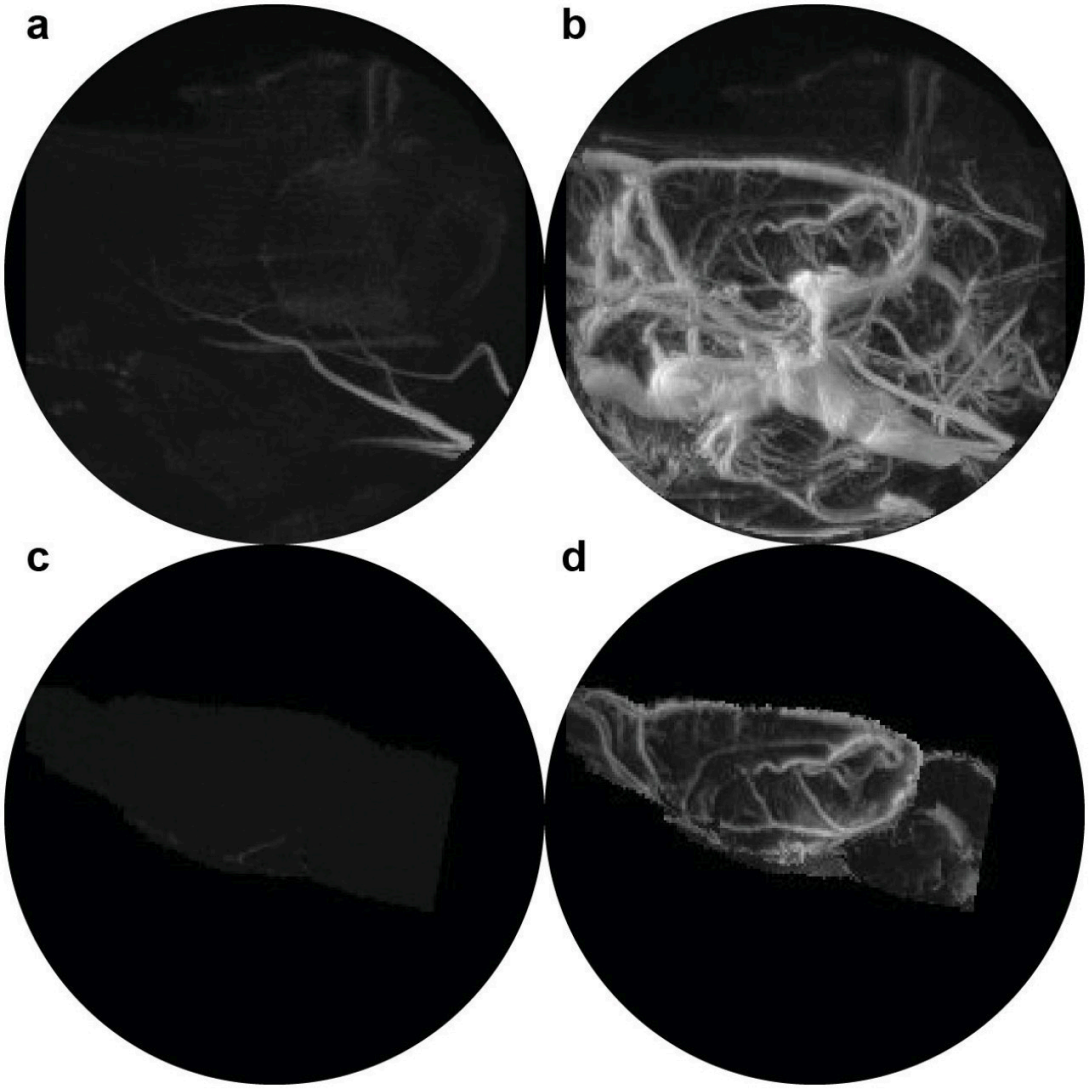
QUTE-CE MRI angiograms. Maximum intensity projection images (MIPs) of the whole rat head at 8 months are displayed (a) pre-contrast and (b) post-contrast 14mg/kg ferumoxytol. Unique contrast-enhanced vascular MIPs are obtained, and segmentation of the brain from the pre- and post-contrast images (c and d, respectively) demonstrates that time-of-flight signal enhancement is limited to the arteries at the periphery of the field of view and so the signal does not contribute to biomarker measurements.

Images were obtained using a Bruker Biospec 7.0 T/20 cm USR horizontal magnet (Bruker, Billerica, MA, USA) equipped with a 20-G/cm magnetic field gradient insert (ID = 12 cm, Bruker). On the day of imaging, rats were anesthetized with 1–2% isoflurane, fixed with a tail-vein catheter, and secured into a custom-built restraining system with a quadrature transmit/receive volume coil with 3cm diameter (S2 Fig). During the scanning session (S3 Fig), rats were given an i.v. bolus of about 14 mg/kg Fe (roughly 2 times the clinical dose), adjusted for each rat to produce an initial blood concentration of 200 μg/ml Fe, assuming 7% blood by body weight. Three averages pre- and post-contrast were obtained with high signal-to-noise ratios (SNR). As seen in Fig 1, The QUTE-CE method creates snapshots of all cerebral arterial and venous vasculature, independent of flow velocity, vessel orientation, or arterial or venous route. Pre-contrast images differentiate few anatomical features, except for time-of-flight effects limited to the arteries at the periphery of the image, prior to magnetization reaching steady state from the hard pulse.

3D UTE MRI acquisition parameters for all scans were consistent. Parameters time-to-echo (TE)/repetition time (TR)/flip-angle (FA) = 13 *μ*s/4 ms/20°, high RF pulse bandwidth (BW) block pulse at 200 kHz, 3×3×3 cm^3^ field of view (FOV), 200×200×200 matrix size, and 125,456×3 radial projections to produce 150 *μ*m^3^ isotropic resolution images, with a total scan time of 25 minutes and 5 seconds for three averages.

### 2.3 Analysis

As previously described [28], the blood volume under steady-state conditions for each voxel (Eq 3) was segmented into 173 anatomically distinct regions using a 3D MRI Rat Brain Atlas © (Ekam Solutions LLC, Boston, MA). From these measurements, two separate structural biomarkers were obtained: the statistical mean per region, as the quantitative calculation (QC) of cerebral blood volume (QC-CBV); and second, the average blood volume in voxels filled primarily with small vessels (QC-SVD). This measure of the microvascular density was obtained by fitting the QC-CBV histogram distribution using a bin size 0.05 QC-CBV fraction and fitting a gaussian around the mode (max of histogram) using a width of 0.10 using the MATLAB function PeakFit [28,34]. This regional mode is considered to reflect small vessel density, given the resolution and neuroanatomical structure of the global vascular distribution. In this study, we focused on the microvasculature, but an analysis on the QC-CBV of each neuroanatomical region can be found in S1 Table.

Using software developed at Northeastern University Center for Translational Neuroimaging, in collaboration with Ekam Imaging, the 173-region atlas was custom-fit to each rat dataset using an affine transformation of the atlas to image space. Once the atlas was co-registered to the images, custom MATLAB code was used to analyze individual brain regions.

We conducted a mixed-design ANOVA to analyze the state of the vasculature between genotypes. Next, we performed our planned pairwise comparisons on each brain region in SPSS version 27. We made corrections to control false positives by applying the two-stage step-up method of Benjamini, Krieger and Yekutieli to each family of hypotheses with the false discovery rate set to 0.05 [35] %. This correction was selected over more conservative methods, such as Bonferroni, in order to mitigate significant increases in type-2 error, while still giving control on the amount of type-1 error present [36]. All multiple comparisons correction was performed in GraphPad prism version 8. Power analysis a priori demonstrated that the appropriate data size that can be used to draw our conclusions is 4 animals per group with an alpha 0.05 and power of 85%.

Each intensity image was corrected for B1^-^ field inhomogeneity along the z-axis with data from a homogenous copper sulfate liquid phantom in a 10ml tube (S4 Fig) and motion aligned spatially using the MATLAB SPM12 toolbox developed at UCL (http://www.fil.ion.ucl.ac.uk/spm/) using nearest neighbor interpolation to preserve original intensity values. The intensity will vary due to B1 inhomogeneity, and therefore both the coil sensitivity (B1^-^) and the flip-angle distribution (B1^+^) should be measured throughout the image volume. Therefore, some limitations exist with the current study that are discussed in detail in the Discussion.

QUTE-CE MRI is the combination of acquisition with an optimized 3D UTE pulse sequence to produce a quantitative signal and intra-vascular contrast agent (CA) to selectively enhance the vascular compartment. *B*_1_ field correction is implemented to render a highly quantitative vascular-specific signal throughout the imaging volume [27,28]. The QC-CBV is calculated using a two-volume blood-tissue model. The two-volume model assumes that the measured voxel intensity, *I*_*M*_, is a linear combination of contributions from the blood and tissue compartments. Thus, the measured signal intensity in each voxel is given by,

$$I_{M}=f_{B}\;I_{B}\;+\;f_{T}\;I_{T}$$ (1)

where *I*_*B*_ is the blood intensity, *I*_*T*_ is the tissue intensity, *f*_*B*_ is the voxel fraction of blood, and *f*_*T*_ is the voxel fraction of tissue. The measured change in intensity between any two scans is given by,

$$\Delta I_{M}=f_{B}^{\prime }I_{B}^{\prime }-f_{B}I_{B}+f_{T}^{\prime }I_{T}^{\prime }-f_{T}I_{T}$$ (2)

By selecting a CA confined to the blood plasma, it is possible to vary *I*_*B*_ with intravascular contrast without changing *I*_*T*_, such that $I_{B}^{\prime }\neq I_{B}$ but $I_{T}^{\prime }\approx I_{T}$, where prime denotes the measured intensity after CA injection. The blood fraction after CA injection can be measured as,

$${QC-CBV}_{0}=f_{B_{0}}^{\prime }=\frac{I_{M_{0}}^{\prime }-I_{M}}{I_{B_{0}}^{\prime }-I_{B}}$$ (3)

Where *I*_*M*_ is the pre-contrast image intensity and $I_{M_{0}}^{\prime }$ is the post-contrast image before neurofunctional modulation. *I*_*B*_ and $I_{B_{0}}^{\prime }$ are the B_1_-corrected blood intensity values pre- and post-contrast, respectively. *QC* − *CBV*_0_ is therefore a measure of the steady state partial blood volume used for structural biomarkers.

To determine if there are changes in QC-CBV across different neurological states (e.g., 5% CO_2_ administration), then the need for multiple pre-contrast images in the corresponding state is avoided by using the calculated $f_{B_{0}}^{\prime }$, such that the time-varying QC-CBV thereafter can be written as,

$$QC-CBV\left(t\right)=f_{B}^{\prime }\left(t\right)=\frac{I_{M}^{\prime }\left(t\right)-I_{M_{0}}^{\prime }+f_{B_{0}}^{\prime }\left(I_{B_{0}}^{\prime }-I_{T_{0}}\right)}{I_{B}^{\prime }\left(t\right)-I_{T_{0}}}+\frac{I_{T}^{\prime }\left(t\right)-I_{T_{0}}}{I_{B}^{\prime }\left(t\right)-I_{T_{0}}}\approx \frac{I_{M}^{\prime }\left(t\right)-I_{M_{0}}^{\prime }+f_{B_{0}}^{\prime }\left(I_{B_{0}}^{\prime }-I_{T_{0}}\right)}{I_{B}^{\prime }\left(t\right)-I_{T_{0}}}$$ (4)

Here, the subscript “_0_” represents values during the first post-contrast image. The second term in Eq 4 is ≈ 0 because $I_{T}^{\prime }\left(t\right)-I_{T_{0}}\ll I_{B}^{\prime }\left(t\right)-I_{T_{0}}$, since the tissue compartment is not enhanced by CA unless the blood brain barrier (BBB) is compromised, in which case an increased intensity would reflect CA deposition [31]. Eq 3 is applied to calculate $I_{T_{0}}$.

Blood intensity values were measured along a large vessel, the superior sagittal sinus (SSS). The region-of-interest (ROI) identified post-contrast using a rough ROI followed by selection of maximum intensity voxels along the SSS (1 per slice) and the average value per image was used (S5 Fig). All pre-contrast *I*_*B*_ values obtained with this method can be found in S6 Fig and post-contrast blood values for 8 months S7 Fig.

## 3. Results

Vascular density was analyzed from 173 distinct anatomical regions of interest drawn from atlas-based maps of rat neuroanatomy. We determined significance for QC-SVD for each region, compared to controls. The genotype had a statistically significant effect on QC-SVD F(1,9) = 17.141, p = .002.

**Fig 2:**
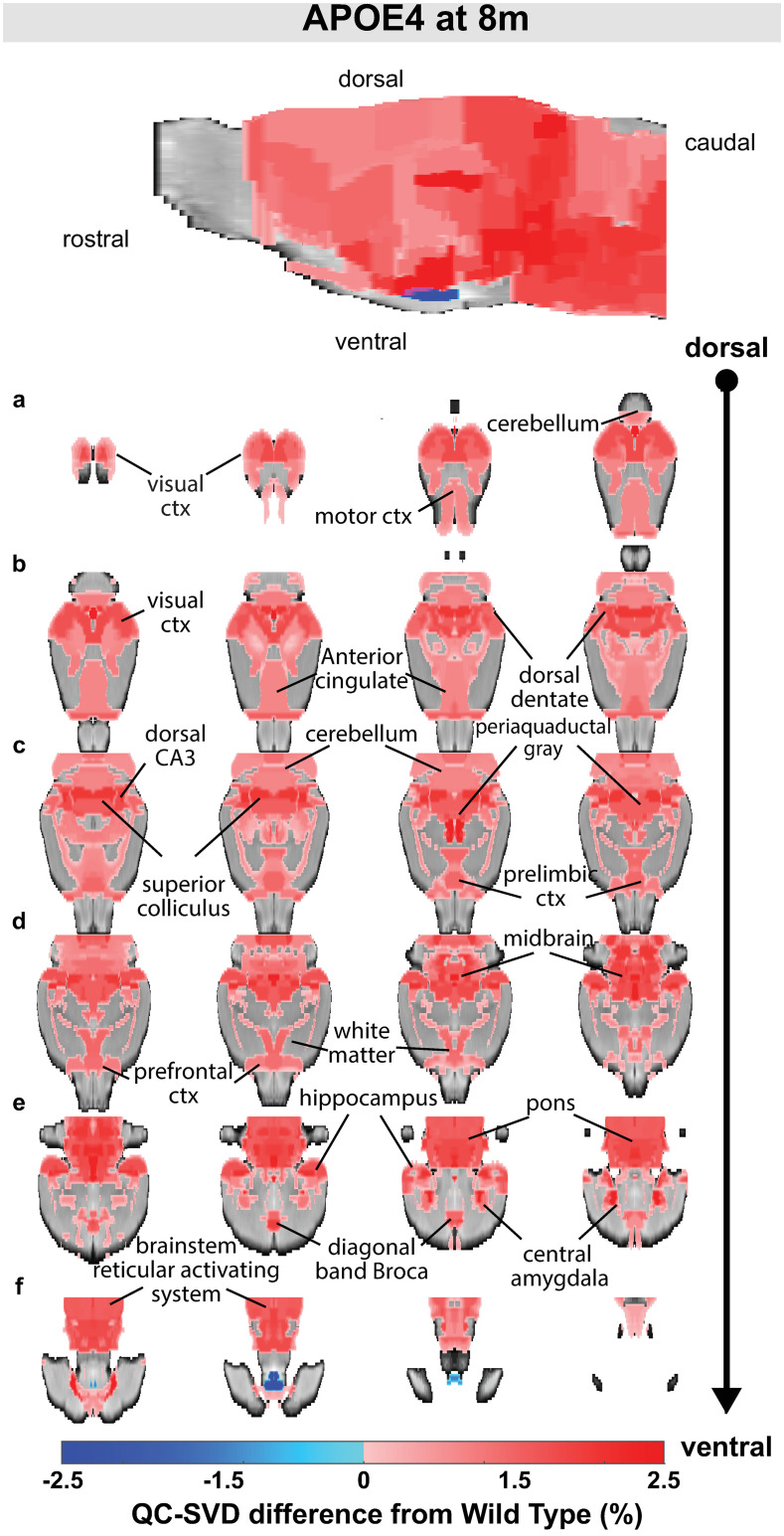
Overview of QC-SVD abnormality detected in APOE4 animals (*q* < .05). Measurements were collected at 8 months. When significant, the differences from WT are reflected in the color scale (hyper-vascularized = red, hypo-vascularized = blue). The brain volume affected was 48.3% hyper-vascularized and <0.1% hypo-vascularized. Sagittal maximum intensity projections are displayed on top. Rows labeled (a-f) are coronal brain slices progressing dorsal to ventral with left-to-right ordering. Regions are labeled. An anatomical atlas with 174 regions was used for region-of-interest selection.

Analysis of the small vessel density with the QC-SVD biomarker revealed abnormal increases in microvascular density at 8 months in 48.3% of the brain volume (75 regions). Less than 0.1% of the brain showed significant decreases in QC-SVD (2 regions). Fig 2 displays these changes, rendered in both 2D coronal slices and 3D MIPs; red and blue denote areas of hyper- and hypo-vascularity, respectively. The brainstem reticular activating system comprising the gigantocellularis, solitary tract nucleus, parabrachial nucleus, parvicellular reticular area, principle sensory nuclei, and dorsal paragigantocellularis are hyper-vascularized (Fig 2 row f). This ascending activating system extends into the pons, with the pontine reticular nuclei and midbrain/thalamus, and the midbrain reticular nucleus and raphe.

**Fig 3:**
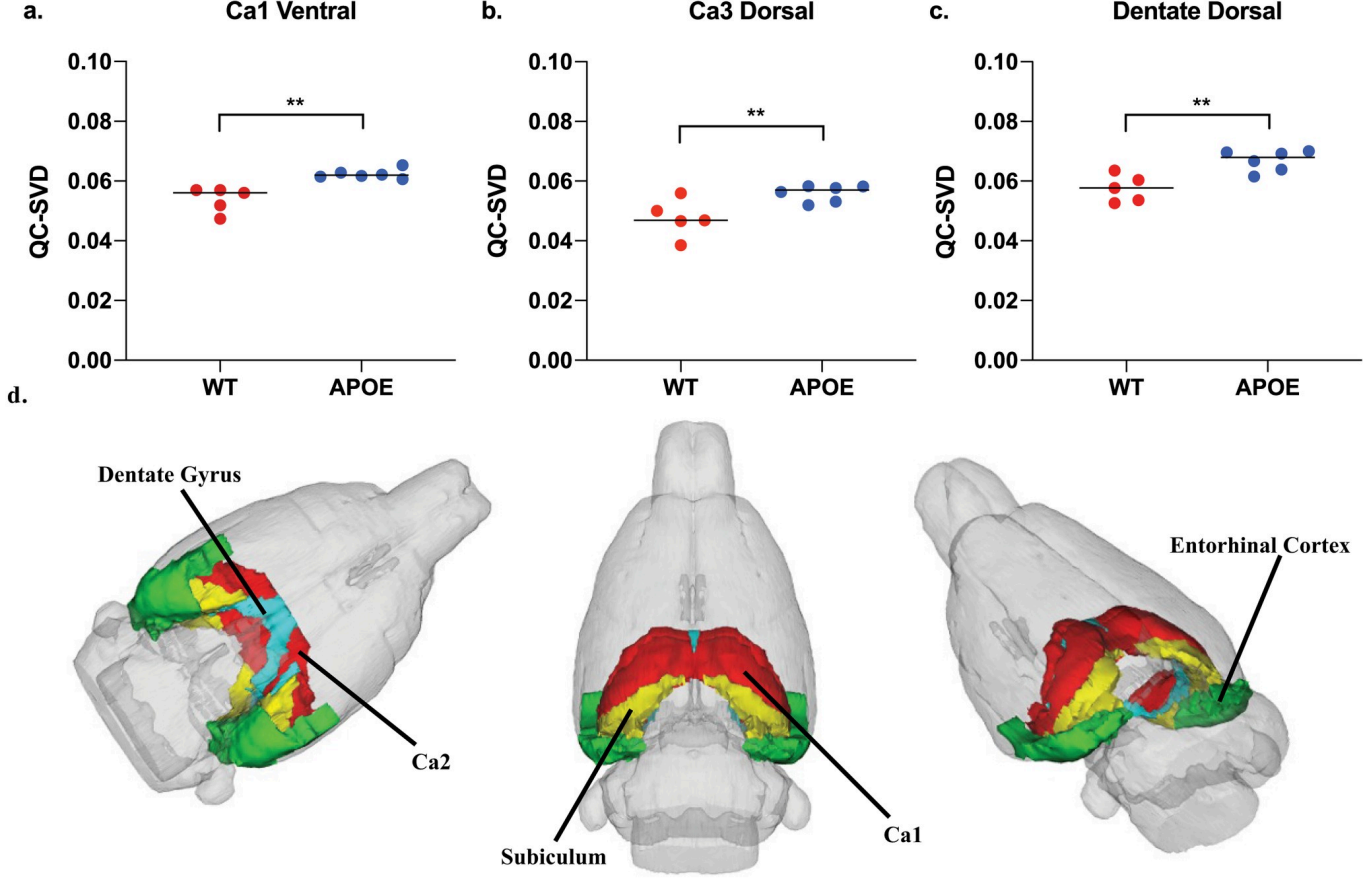
Tri-synaptic circuit. (a-c) Represents the 3 core regions associated with the hippocampal tri-synaptic circuit involved in learning and memory formation (d) A neuroanatomical visual display of each of these regions in the tri-synaptic circuit. The subiculum and entorhinal cortex are also displayed here, as both of these regions are associated with the function of this circuit. (* p<0.05, ** p<0.01).

**Fig 4:**
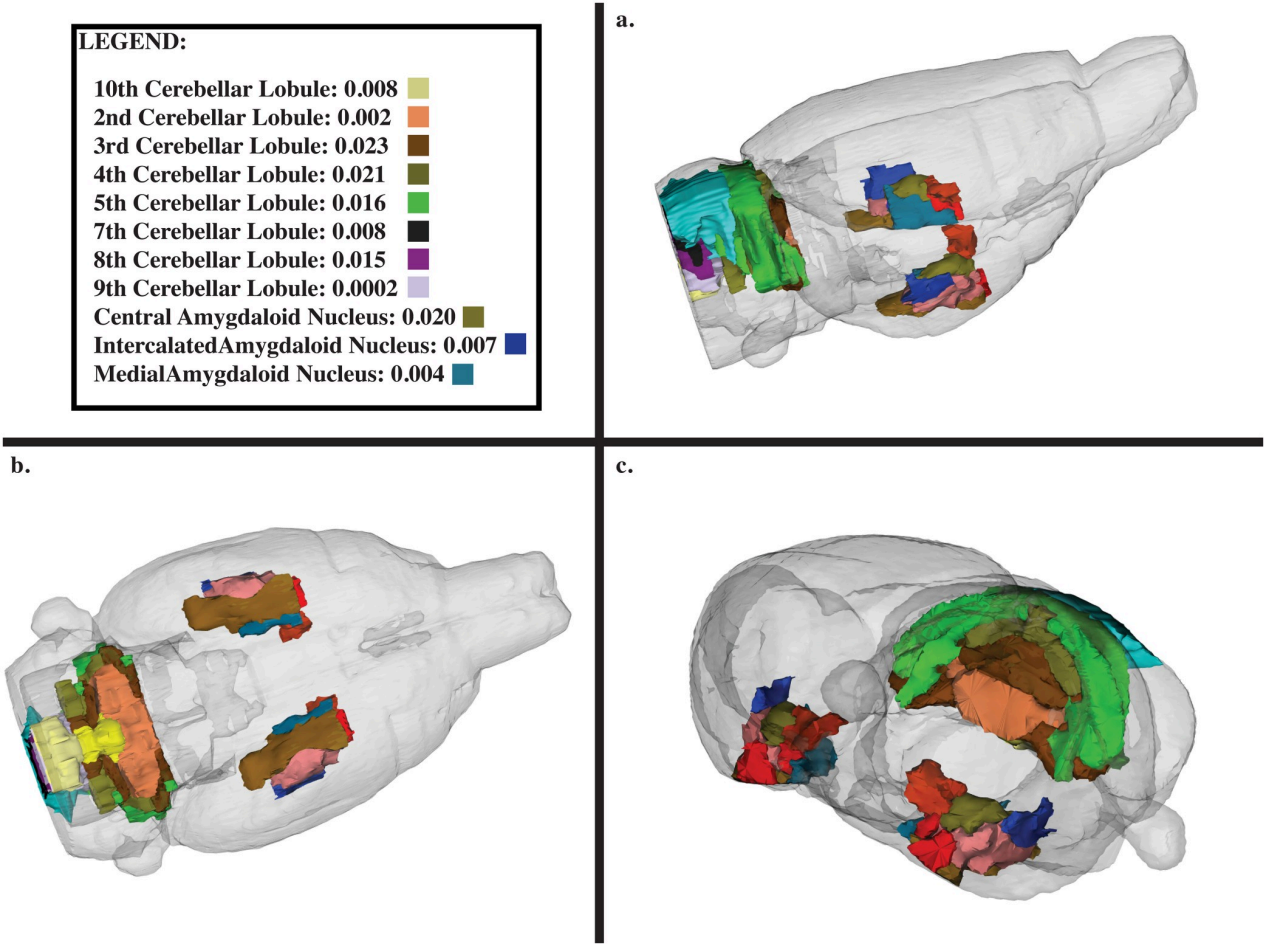
Neuroanatomical display of the amygdala and 10 cerebellar lobules. (a) Side profile view of rat brain. (b) ventral view of rat brain. (c) Side profile view of rat brain of anterior rostral brain. Legend displays only regions that were statistically significant, with both the p-value and the corresponding color represented on the 174-region neuroanatomical atlas.

**Fig 5:**
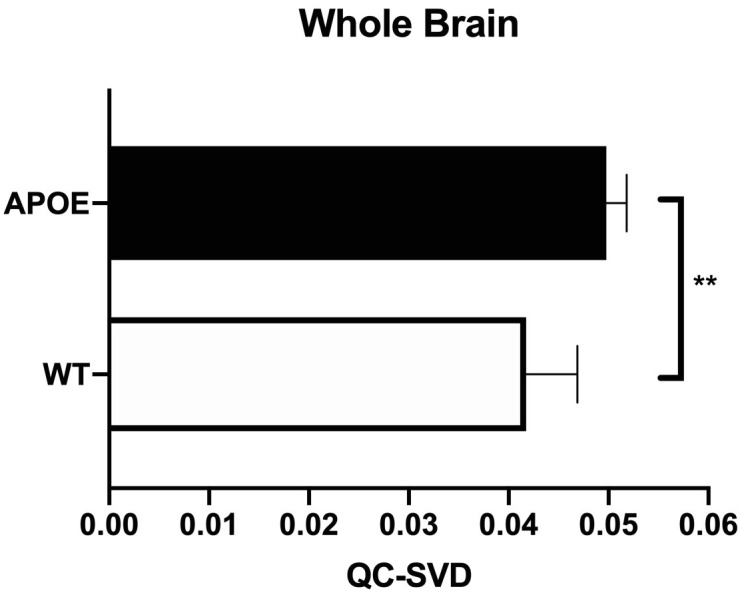
Average whole brain QC-SVD. All of the voxels in the entire rat brain were sampled as one single region-of-interest to calculate the QC-SVD of the whole brain of each animal and compared between genotypes. An independent t-test (p = 0.002) was utilized for this additional comparison. This illustration includes the average QC-SVD (p = 0.003) and SD between genotypes. Group sample sizes at 8 months were (WT, n = 5; APOE4, n = 6).

Additionally, the hippocampal complex, which has major implications in learning and memory, was severely affected. The impacted neuroanatomical regions in this complex included the dentate gyrus, Cornu Ammonis 1 (CA1), and Cornu Ammonis 3 (CA3), which all have markedly increased QC-SVD as displayed in Fig 3. See S10 Fig to see the effect on the entorhinal cortex and subiculum, both regions that are associated with the functioning of the tri-synaptic circuit. Additionally, the amygdala and cerebellar lobules had increased QC-SVD. These anatomical regions have implications on conditioned learning and processing procedural memories, respectively. The corresponding p-values are depicted in Fig 4. At the whole brain level, the microvascular volume across all voxels in the brain for APOE4 animals (0.05 QC-SVD) was significantly higher than in wildtype animals (0.04 QC-SVD) in this study (Fig 5). These results indicate a global state of hyper- micro-vascular volume at 8 months in the APOE4 model. S1 Table displays a full list of both affected, and unaffected, regions in this dataset.

## 4. Discussion

We hypothesized that the biomarkers would reveal site-specific vascular abnormalities associated with APOE4 in the rat model. We began with one key assumption: microvascular density would be remodeled as necessary to match cerebral metabolic demands for glucose and oxygen [37,38].

This APOE4 knock-in rat model has been shown to have greater resting state functional connectivity at 6-months of age as compared to WT controls using standard fMRI methods in areas involved with cognition, arousal, and emotion [39]. The increased microvascular density described here might be a neuroadaptive response to a metabolic dysfunction. At six months of age these APOE4 females show no loss of cognitive function [39]. Interestingly, asymptomatic female Ɛ4 carriers ages 20–35 show increased functional connectivity as compared to controls [13].

### 4.1 Vascular abnormality

At 8 months, approximately 22 human years [32], we detected increased signal in 48.3% of the brain associated with the APOE4 phenotype (q<0.05, multiple comparisons). Our supplemental exploration including large vessels demonstrated a similar, but far less pronounced increases (18.8% of the brain for p<0.05 with no correction). Researchers increasingly recognize small vessels as important pathophysiological indicators in ADRD development [8,40]. Old APP23 mice present with disrupted blood flow and abnormalities in microcirculatory architecture, including angiogenesis with disease progression [41]. APP23 mice also have increased vascular density associated with amyloid plaques [42]. Even studies in cerebral ischemia show a pattern of newly formed vessels at the border of infarcts within 2 to 3 days [43]. Tau models of AD also present with small vessel abnormalities and increased small vessel density in the cortex and elsewhere. Interestingly, microvessel density has been found to increase from twelve to fifteen months but decline thereafter in one rodent model [44].

Our results at 8 months of age support the findings of early hyper-vascularization. It is possible that this early increased small vessel density may serve as a compensatory mechanism for vascular dysfunction within the neurovascular unit, such as reduced vasodilatory reactivity [45] or impaired molecular signaling to pericytes [6]. Further studies should be performed to examine these possibilities. Recently, Ferris et al., found that six-month old APOE4 rats of the model used here exhibited hyper-connectivity, which is consistent with our findings. An increased microvascular density could explain the increased BOLD signal found in young human carriers both at rest and during memory tasks, despite having lower CO_2_-induced vascular reactivity [46]. Ostergaard suggested that brain capillary dysfunction underlies the development of a neuronal energy crisis which triggers AD due to reduced capillary transit time [47,48]. While the molecular and cellular mechanisms for neurovascular dysfunction and related compensation remain mostly unknown at present for APOE4 carriers [49] a denser microvascular network—one with more red blood cells and surface area for oxygen exchange—could explain the dramatic BOLD signal response found in the fMRI signal of young APOE4 carriers. Finally, it is important to acknowledge the possibility that this trend eventually reverses towards hypo-vascularization, which is more commonly associated with neurodegeneration [50].

Additionally, the role of blood -brain -barrier (BBB) leakage as a possible contributor to these findings is one of great interest. Literature suggests that BBB leakage is positively correlated to aging, cognitive decline and AD in APOE4 humans [51] BBB leakage was reported in 6-month-old ApoE4 mice of unknown gender [52], but it is hard to determine the implications for our study on female APOE4 rats. Female rodents are known to progress differentially than males in APOE4-associated pathology, and a study by Ferris et al, it was found that these APOE4 females were not cognitively dysfunctional at 6 months [39]. Retrospectively considering the possibility of BBB leakage modulation on the QUTE-CE signal, we performed a small study on available rats (n = 5; APOE4 16-month-old (~40 human years) females; see S11 Fig). These animals were on a high fat diet, and older, both of which would potentially exacerbate any leakage. Though, even at this later stage there was still no indication of BBB leakage in this animal model with QUTE-CE MRI using previously described methods [31]. These findings suggest that it is less likely that BBB leakage had a significant contribution to our measurements in 8-month-old APOE4 animals. Even with this in consideration, further exploration with histological analysis is warranted to adequately determine the biological cause of the detected vascular abnormality.

### 4.2 Limitations of this study

This is a small cross-sectional study; however, we were still able to identify key differences in the APOE4 model due to robust modulations in a quantitative signal. Another limitation here is that it is possible that additional abnormality was present but not detected with p-value tests. On Bruker scanners, we expect that signal-to-noise ratio (SNR) may be augmented by 4 or 8-fold with current available commercial hardware (phased array surface head coil and cryoprobe, respectively). The best combination of hardware for this experiment would be a surface coil, for high sensitivity, combined with a large volume excitation coil, for robust mapping of B1^-/+^ fields. In this study, coil inhomogeneity in the x-y plane was ignored in the volume coil because it is rather homogenous. The flip-angle was not measured, which is important for quantitative measurements, but B1^+^ inaccuracy resulting from this comparison will be minimal due to geometric consistency (S1, S2 and S4 Figs). Sex is a limiting factor in this study; this study focused only on female rats, however, more than 60% of humans afflicted with AD and other dementias over the age of 65 are women. Likewise, the Ɛ4 allele is, first, found more often in women with AD than men [53]; second, associated with an earlier age of familial AD onset [54]; and, third, presents an overall greater risk for developing AD [55].

### 4.3 Implications towards clinical translation

We have investigated the use of QUTE-CE MRI biomarkers for ApoE-Ɛ4 (APOE4) induced vascular abnormality in a knock-in (TGRA8960) female rat model. Our results suggest that quantitative assessment of vasculature is important to evaluate brain health. As research interest has been growing towards vascular causes of ADRD, QUTE-CE MRI is readily translatable to the clinic and could prove to be a uniquely important tool in non-invasive studies of vascular pathogenesis.

## 5 Conclusion

We present measurements of neurovascular imaging biomarkers of the microvasculature using QUTE-CE MRI at an early time-point of 8-months in an APOE4 human knock-in rat model compared to littermate controls. Our results indicate that vascular abnormality can be detected with high sensitivity, providing new insights into disease pathophysiology. Here, an increased small vessel density was found resulting from APOE4. Future efforts should be taken to analytically characterize the biomarkers, both preclinically and clinically, to determine effect size and for validation for human use in precision medicine, and to rigorously examine the structural, functional and BBB leakage contributions.

## Supporting information

S1 Fig Animal weights and brain volumes. (a) Weights and (b) brain volume at the time of MRI measurement. Brin volume was measured from the number of voxels fit into the anatomical atlases. Volume is displayed in ml (left axis) and number of voxels in the brain from 3D UTE images (right axis). There was no statistically significant difference in APOE4 weight or brain volumes compared to the WTs (p>0.05).
(DOCX)

S2 Fig MRI rat coil and mechanical restraint setup. Each animal was restrained within a quadrature volume coil by using a mouth bar to lock the jaw in place. 1–2% isoflurane was used to anesthetize each animal before securing it within the body tube using shoulder pins as well as neck and nose presses; this minimized any movement from the animal during the scanning procedure. Finally, the animals were secured into a chassis and fit with an intravenous tail-vein catheter before being placed into a 7T Bruker Scanner where the QUTE-CE method was applied.
(DOCX)

S3 Fig MRI scan procedures. 8-month-old animals were scanned as depicted, with each 25-minute 3D UTE scan performed by averaging three 8m22s scans to improve signal-to-noise. Ferumoxytol was injected after pre-contrast imaging and post-contrast scanning began approximately 1 minute after flushing the bolus.
(DOCX)

S4 Fig B1- correction with cylindrical phantom. A 10-mL tube filled with 5mM copper sulfate (the phantom) was used for B1^-^ correction. The phantom was centered in the X-Y plane of coil and a 3D UTE scan was performed using the same experiment rat scan protocol. (a) The phantom and the region of interest are visualized in three orthogonal planes. From left to right: axial, sagittal, coronal. (b) The intensity was measured along the z-axis (sagittal slice) to correct for the B1^-^ inhomogeneity, also known as the coil sensitivity profile. A 2^nd^ degree polynomial was fit to the data and this fitting function was used to normalize all 3D UTE images (both pre- and post-contrast images) for coil sensitivity. The XY inhomogeneity within rat brains was ignored in this because of the minimal variance within the area being investigated in our study.
(DOCX)

S5 Fig Drawing blood intensity regions of interest in the superior sagittal sinus. Rough 3D segmentations of the superior sagittal sinus were made in 3DSlicer as illustrated in the image. MATLAB was utilized to multiply the 3D UTE image by the label map, and to take the highest intensity voxel along the Superior Sagittal Sinus.
(DOCX)

S6 Fig Pre-contrast blood intensity values at 8m. Pre-contrast intensity values measured in the superior sagittal sinus (SSS) at the 8m time-point, note that the intensity for pre-contrast blood is similar in all animals, which is expected since the signal is T1-dependent. Standard deviations represent signal variation along the SSS for each animal.
(DOCX)

S7 Fig Post-contrast blood intensity values at 8m. Post-contrast blood intensity values as measured in the superior sagittal sinus (SSS) and standard deviations represent signal variation along the SSS for each animal.
(DOCX)

S8 Fig Individual animal data for QC-SVD abnormality at 8m (p<0.05). (a) QC-SVD for microvasculature organized from left to right in decreasing p-value from p = 2E-6 to p = 0.004 and continued in (b) for p = 0.004 to p = 0.047.
(DOCX)

S9 Fig Individual animal data for QC-SVD abnormality at 8m (p<0.05) continued. QC-SVD for microvasculature for regions containing at least one measurement of QC-SVD > 0.1, organized from left to right in decreasing p-values from 0.0002 to 0.0360.
(DOCX)

S10 Fig Additional regions associated with tri-synaptic circuit function. The subiculum and Entorhinal cortex were both highly impacted in the APOE4 rat model. Statistically analyses were performed with ANOVA.
(DOCX)

S11 Fig BBB leakage assessment. In order to confirm that blood-brain barrier leakage was not a confound in our experiment, we later assessed available APOE4 rats (n = 5) that were slightly older (16 months) and on a high fat diet- both of which would exacerbate BBB leakage if present. (a) The average QC-SVD is displayed when sampling the entire rat brain over time. (b-g) QC-SVD over time for various regions associated with the hippocampus. At 16 months of age. no visible BBB leakage was detected, and this was confirmed (p>0.05) with a linear regression model, testing for an increase in slope over the first 5 scans at the whole brain level.
(DOCX)

S1 Table HFD BBB leakage assessment summary. Linear regression analysis was performed to check if any region had a statistically significant increase in slope over 5 scans, which is an indicator for BBB leakage. Across 5 APOE animals, only 1 region (gigantocellular reticular nucleus, 1.07% brain volume) of 1 animal indicated a statistically significant increase in signal over time.
(DOCX)

S2 Table (XLSX)
