# Supplementary material for: Neurovascular imaging with QUTE-CE MRI in APOE4 rats reveals early vascular abnormalities
Source: PLoS One. 2021 Aug 27;16(8):e0256749. doi: 10.1371/journal.pone.0256749 (PMC8396782; doi:10.1371/journal.pone.0256749)
Supplement: S1 Fig — (a) Weights and (b) brain volume at the time of MRI measurement. Brin volume was measured from the number of voxels fit into the anatomical atlases. Volume is displayed in ml (left axis) and number of voxels in the brain from 3D UTE images (right axis). There was no statistically significant difference in APOE4 weight or brain volumes compared to the WTs (p>0.05). (DOCX) [file pone.0256749.s001.docx]

**a**


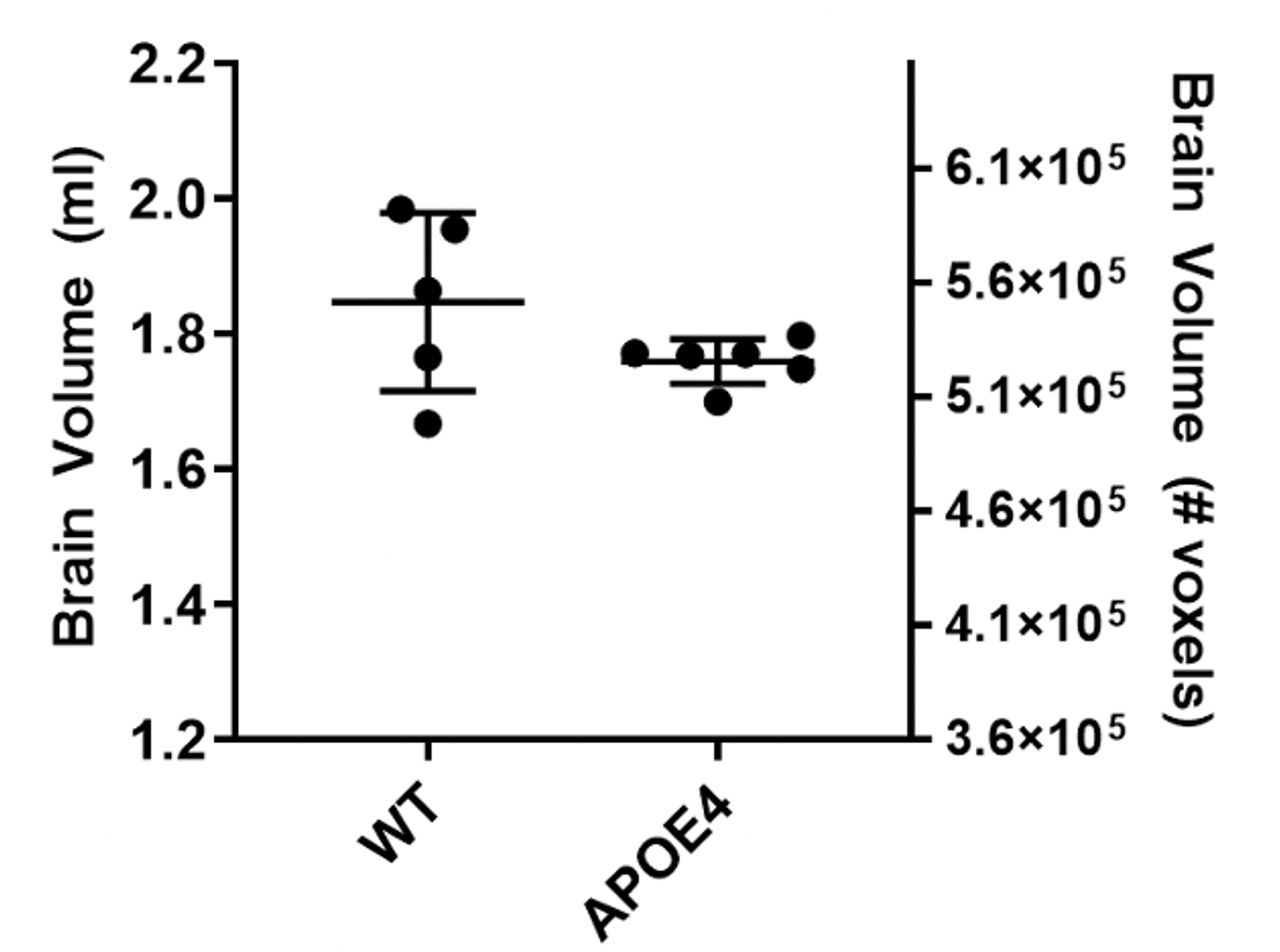


**b**

Supplementary Figure 1. Animal weights and brain volumes. (a) Weights and (b) brain volume at the time of MRI measurement. Brin volume was measured from the number of voxels fit into the anatomical atlases. Volume is displayed in ml (left axis) and number of voxels in the brain from 3D UTE images (right axis). There was no statistically significant difference in APOE4 weight or brain volumes compared to the WTs (p>0.05).
