# Supplementary material for: Neurovascular imaging with QUTE-CE MRI in APOE4 rats reveals early vascular abnormalities
Source: PLoS One. 2021 Aug 27;16(8):e0256749. doi: 10.1371/journal.pone.0256749 (PMC8396782; doi:10.1371/journal.pone.0256749)
Supplement: S2 Fig — Each animal was restrained within a quadrature volume coil by using a mouth bar to lock the jaw in place. 1–2% isoflurane was used to anesthetize each animal before securing it within the body tube using shoulder pins as well as neck and nose presses; this minimized any movement from the animal during the scanning procedure. Finally, the animals were secured into a chassis and fit with an intravenous tail-vein catheter before being placed into a 7T Bruker Scanner where the QUTE-CE method was applied. (DOCX) [file pone.0256749.s002.docx]

**
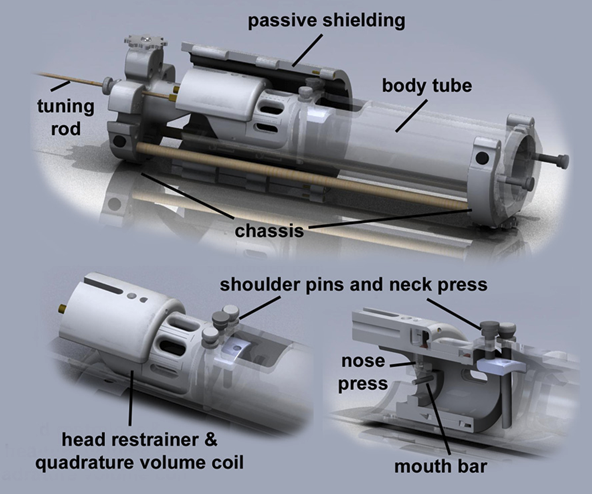
**

Supplementary Figure 2. MRI rat coil and mechanical restraint setup. Each animal was restrained within a quadrature volume coil by using a mouth bar to lock the jaw in place. 1-2% isoflurane was used to anesthetize each animal before securing it within the body tube using shoulder pins as well as neck and nose presses; this minimized any movement from the animal during the scanning procedure. Finally, the animals were secured into a chassis and fit with an intravenous tail-vein catheter before being placed into a 7T Bruker Scanner where the QUTE-CE method was applied.
