# Supplementary material for: Neurovascular imaging with QUTE-CE MRI in APOE4 rats reveals early vascular abnormalities
Source: PLoS One. 2021 Aug 27;16(8):e0256749. doi: 10.1371/journal.pone.0256749 (PMC8396782; doi:10.1371/journal.pone.0256749)
Supplement: S3 Fig — 8-month-old animals were scanned as depicted, with each 25-minute 3D UTE scan performed by averaging three 8m22s scans to improve signal-to-noise. Ferumoxytol was injected after pre-contrast imaging and post-contrast scanning began approximately 1 minute after flushing the bolus. (DOCX) [file pone.0256749.s003.docx]

**
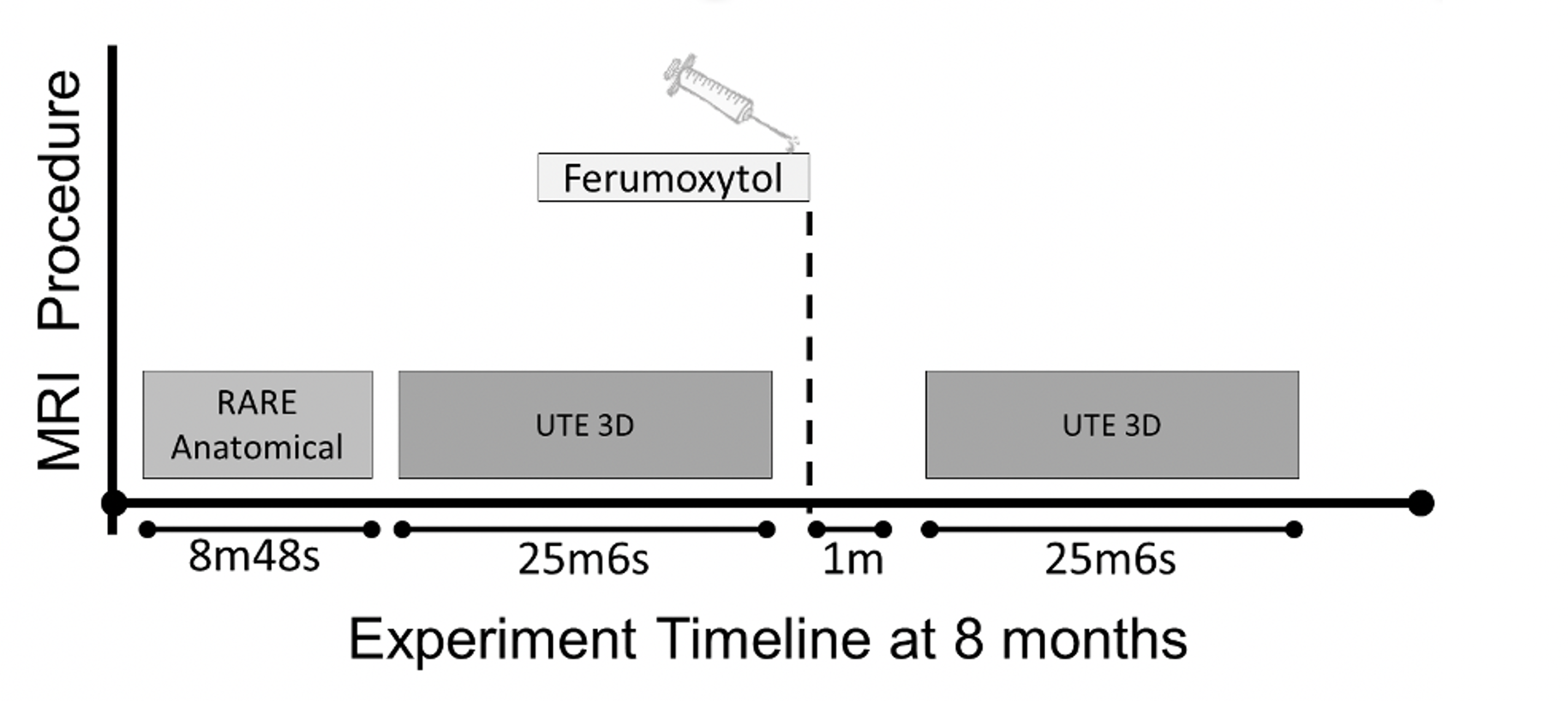
**

Supplementary Figure 3. MRI scan procedures**.** 8-month-old animals were scanned as depicted, with each 25-minute 3D UTE scan performed by averaging three 8m22s scans to improve signal-to-noise. Ferumoxytol was injected after pre-contrast imaging and post-contrast scanning began approximately 1 minute after flushing the bolus.
