# Supplementary material for: Neurovascular imaging with QUTE-CE MRI in APOE4 rats reveals early vascular abnormalities
Source: PLoS One. 2021 Aug 27;16(8):e0256749. doi: 10.1371/journal.pone.0256749 (PMC8396782; doi:10.1371/journal.pone.0256749)
Supplement: S4 Fig — A 10-mL tube filled with 5mM copper sulfate (the phantom) was used for B1- correction. The phantom was centered in the X-Y plane of coil and a 3D UTE scan was performed using the same experiment rat scan protocol. (a) The phantom and the region of interest are visualized in three orthogonal planes. From left to right: axial, sagittal, coronal. (b) The intensity was measured along the z-axis (sagittal slice) to correct for the B1- inhomogeneity, also known as the coil sensitivity profile. A 2nd degree polynomial was fit to the data and this fitting function was used to normalize all 3D UTE images (both pre- and post-contrast images) for coil sensitivity. The XY inhomogeneity within rat brains was ignored in this because of the minimal variance within the area being investigated in our study. (DOCX) [file pone.0256749.s004.docx]

**
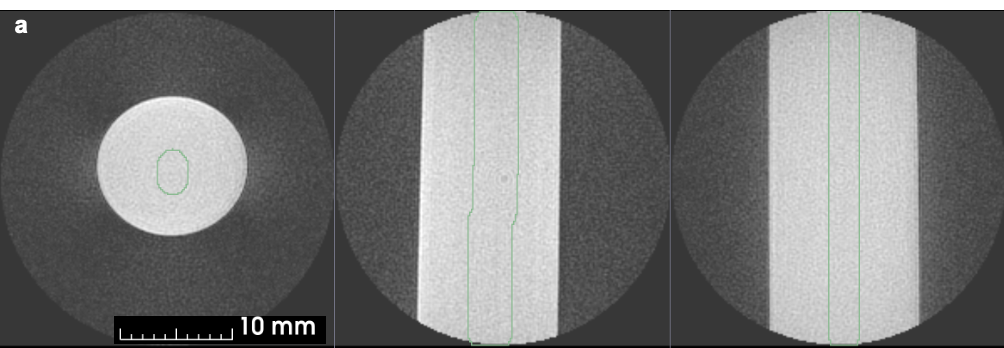
**

**
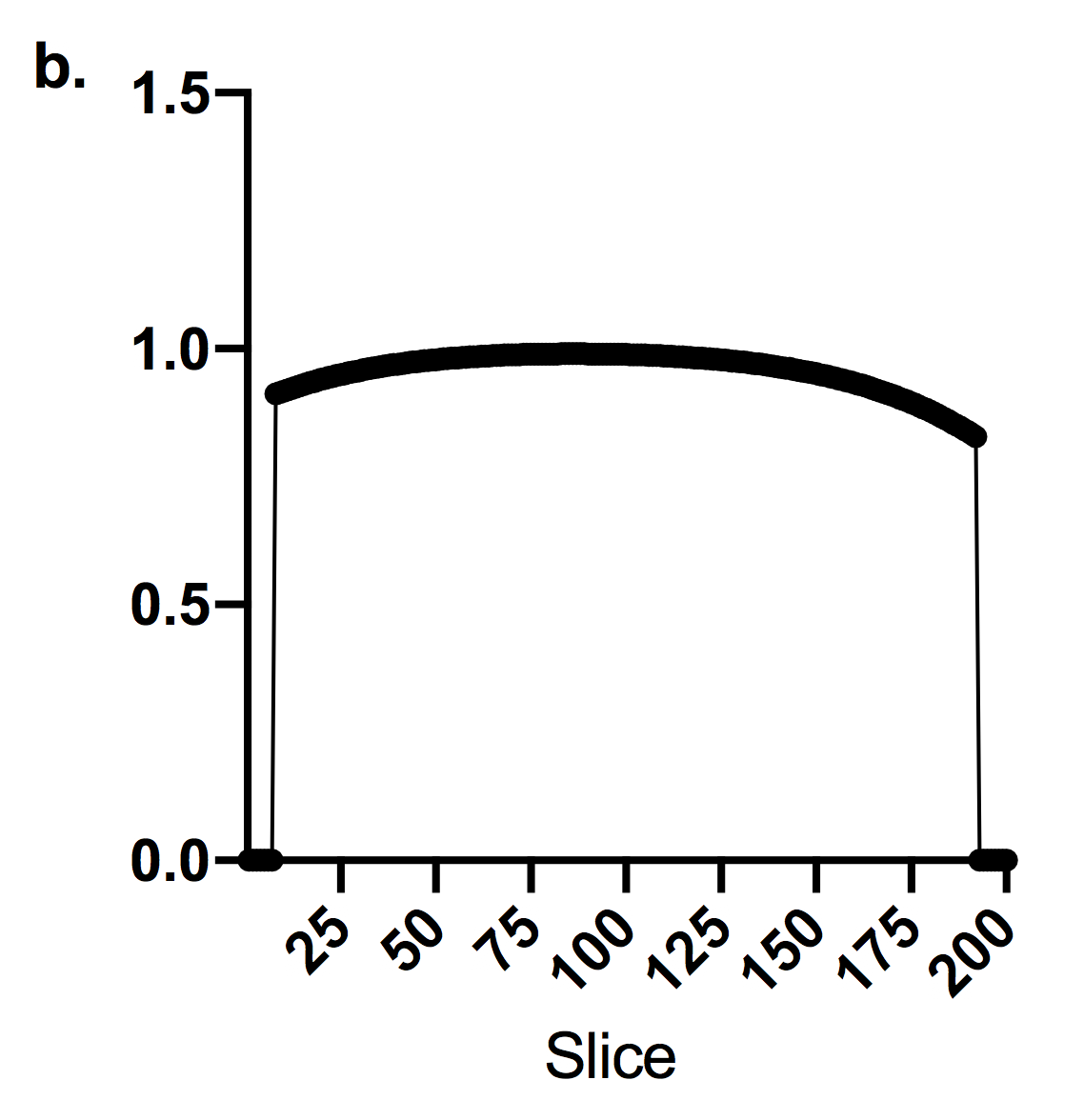
**

Supplementary Figure 4. B1- correction with cylindrical phantom**.** A 10-mL tube filled with 5mM copper sulfate (the phantom) was used for B1^-^ correction. The phantom was centered in the X-Y plane of coil and a 3D UTE scan was performed using the same experiment rat scan protocol. (a) The phantom and the region of interest are visualized in three orthogonal planes. From left to right: axial, sagittal, coronal. (b) The intensity was measured along the z-axis (sagittal slice) to correct for the B1^-^ inhomogeneity, also known as the coil sensitivity profile. A 2^nd^ degree polynomial was fit to the data and this fitting function was used to normalize all 3D UTE images (both pre- and post-contrast images) for coil sensitivity. The XY inhomogeneity within rat brains was ignored in this because of the minimal variance within the area being investigated in our study.
