# Supplementary material for: Neurovascular imaging with QUTE-CE MRI in APOE4 rats reveals early vascular abnormalities
Source: PLoS One. 2021 Aug 27;16(8):e0256749. doi: 10.1371/journal.pone.0256749 (PMC8396782; doi:10.1371/journal.pone.0256749)
Supplement: S5 Fig — Rough 3D segmentations of the superior sagittal sinus were made in 3DSlicer as illustrated in the image. MATLAB was utilized to multiply the 3D UTE image by the label map, and to take the highest intensity voxel along the Superior Sagittal Sinus. (DOCX) [file pone.0256749.s005.docx]

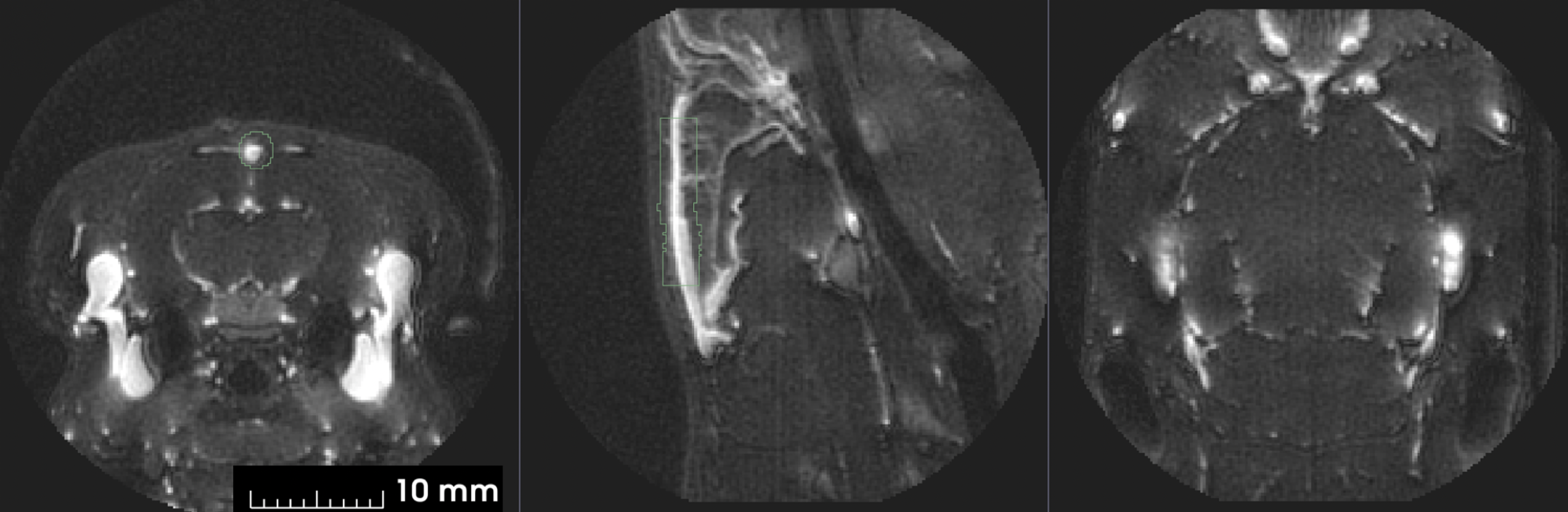


Supplementary Figure 5. Drawing blood intensity regions of interest in the superior sagittal sinus. Rough 3D segmentations of the superior sagittal sinus were made in 3DSlicer as illustrated in the image. MATLAB was utilized to multiply the 3D UTE image by the label map, and to take the highest intensity voxel along the Superior Sagittal Sinus.
