# Supplementary material for: Neurovascular imaging with QUTE-CE MRI in APOE4 rats reveals early vascular abnormalities
Source: PLoS One. 2021 Aug 27;16(8):e0256749. doi: 10.1371/journal.pone.0256749 (PMC8396782; doi:10.1371/journal.pone.0256749)
Supplement: S6 Fig — Pre-contrast intensity values measured in the superior sagittal sinus (SSS) at the 8m time-point, note that the intensity for pre-contrast blood is similar in all animals, which is expected since the signal is T1-dependent. Standard deviations represent signal variation along the SSS for each animal. (DOCX) [file pone.0256749.s006.docx]

**
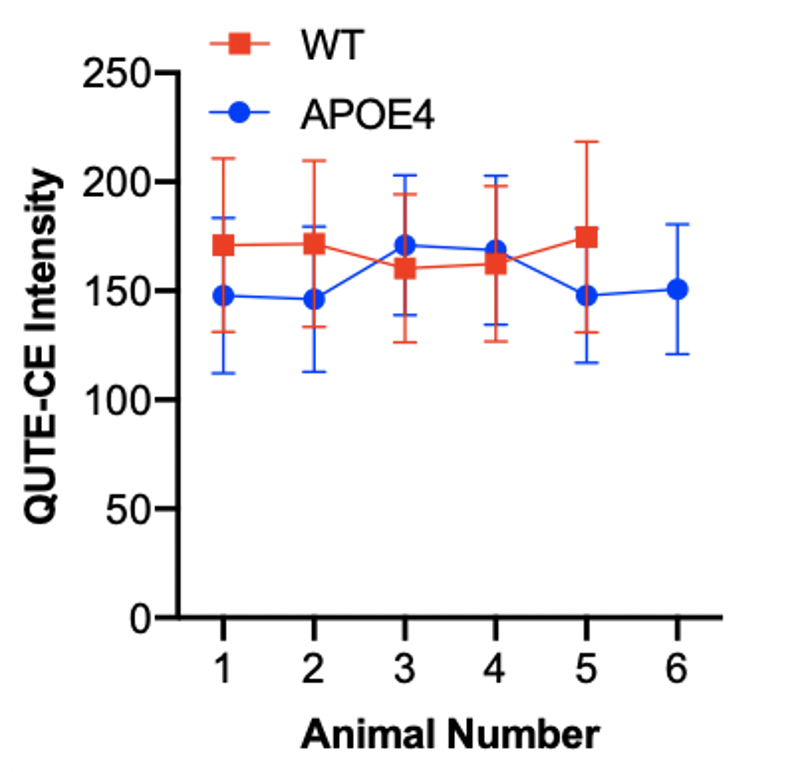
**

Supplementary Figure 6. Pre-contrast blood intensity values at 8m. Pre-contrast intensity values measured in the superior sagittal sinus (SSS) at the 8m time-point, note that the intensity for pre-contrast blood is similar in all animals, which is expected since the signal is T1-dependent. Standard deviations represent signal variation along the SSS for each animal.
