# Supplementary material for: Neurovascular imaging with QUTE-CE MRI in APOE4 rats reveals early vascular abnormalities
Source: PLoS One. 2021 Aug 27;16(8):e0256749. doi: 10.1371/journal.pone.0256749 (PMC8396782; doi:10.1371/journal.pone.0256749)
Supplement: S7 Fig — Post-contrast blood intensity values as measured in the superior sagittal sinus (SSS) and standard deviations represent signal variation along the SSS for each animal. (DOCX) [file pone.0256749.s007.docx]

Supplementary Figure 7. Post-contrast blood intensity values at 8m. Post-contrast blood intensity values as measured in the superior sagittal sinus (SSS) and standard deviations represent signal variation along the SSS for each animal.
