# Supplementary material for: Neurovascular imaging with QUTE-CE MRI in APOE4 rats reveals early vascular abnormalities
Source: PLoS One. 2021 Aug 27;16(8):e0256749. doi: 10.1371/journal.pone.0256749 (PMC8396782; doi:10.1371/journal.pone.0256749)
Supplement: S8 Fig — (a) QC-SVD for microvasculature organized from left to right in decreasing p-value from p = 2E-6 to p = 0.004 and continued in (b) for p = 0.004 to p = 0.047. (DOCX) [file pone.0256749.s008.docx]

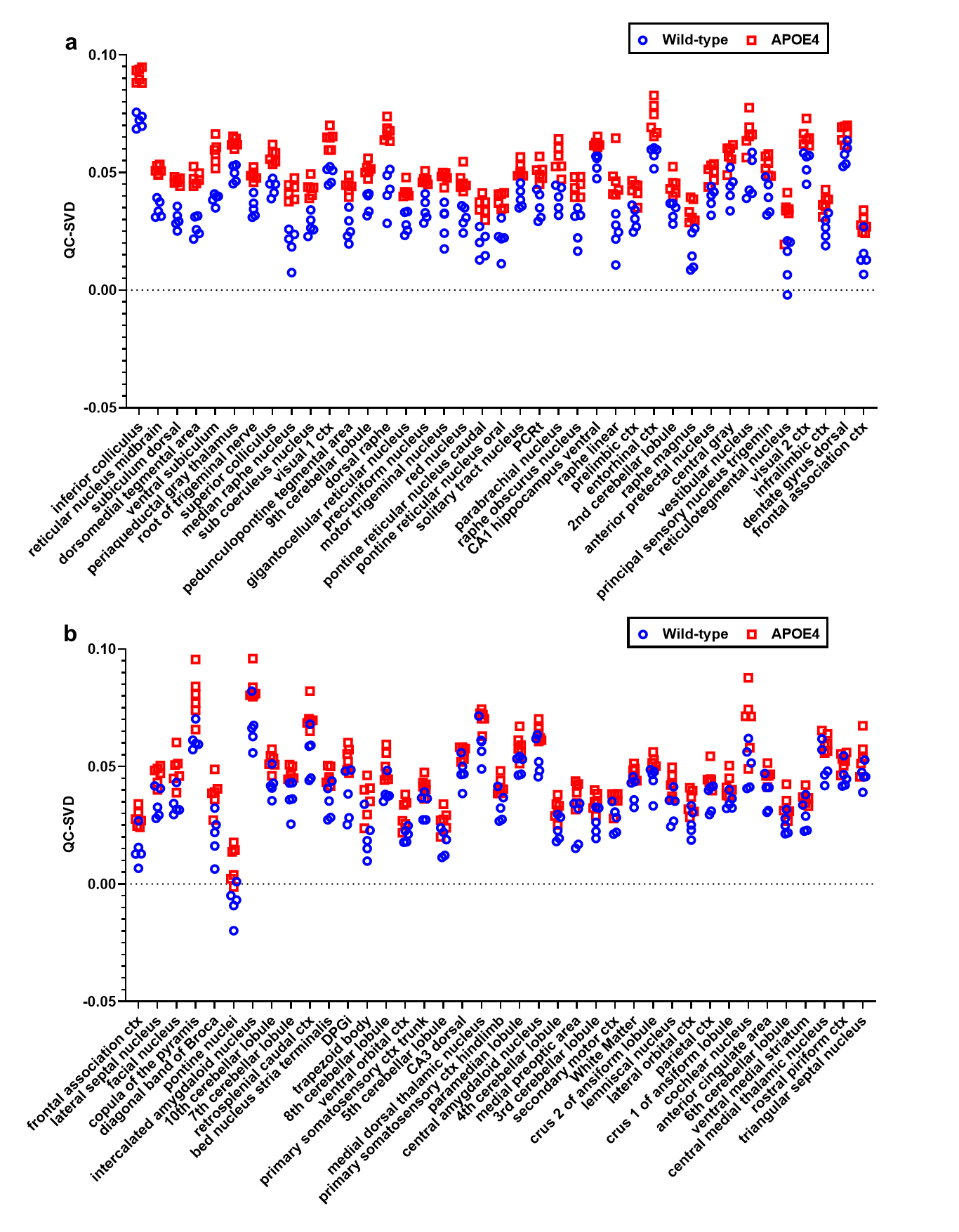


Supplementary Figure 8. Individual animal data for QC-SVD abnormality at 8m (p<0.05). (a) QC-SVD for microvasculature organized from left to right in decreasing p-value from p=2E-6 to p=0.004 and continued in (b) for p=0.004 to p=0.047.
