# Supplementary material for: Neurovascular imaging with QUTE-CE MRI in APOE4 rats reveals early vascular abnormalities
Source: PLoS One. 2021 Aug 27;16(8):e0256749. doi: 10.1371/journal.pone.0256749 (PMC8396782; doi:10.1371/journal.pone.0256749)
Supplement: S9 Fig — QC-SVD for microvasculature for regions containing at least one measurement of QC-SVD > 0.1, organized from left to right in decreasing p-values from 0.0002 to 0.0360. (DOCX) [file pone.0256749.s009.docx]

Supplementary Figure 9. Individual animal data for QC-SVD abnormality at 8m (p<0.05) continued. QC-SVD for microvasculature for regions containing at least one measurement of QC-SVD > 0.1, organized from left to right in decreasing p-values from 0.0002 to 0.0360.
