# Supplementary material for: Neurovascular imaging with QUTE-CE MRI in APOE4 rats reveals early vascular abnormalities
Source: PLoS One. 2021 Aug 27;16(8):e0256749. doi: 10.1371/journal.pone.0256749 (PMC8396782; doi:10.1371/journal.pone.0256749)
Supplement: S10 Fig — The subiculum and Entorhinal cortex were both highly impacted in the APOE4 rat model. Statistically analyses were performed with ANOVA. (DOCX) [file pone.0256749.s010.docx]

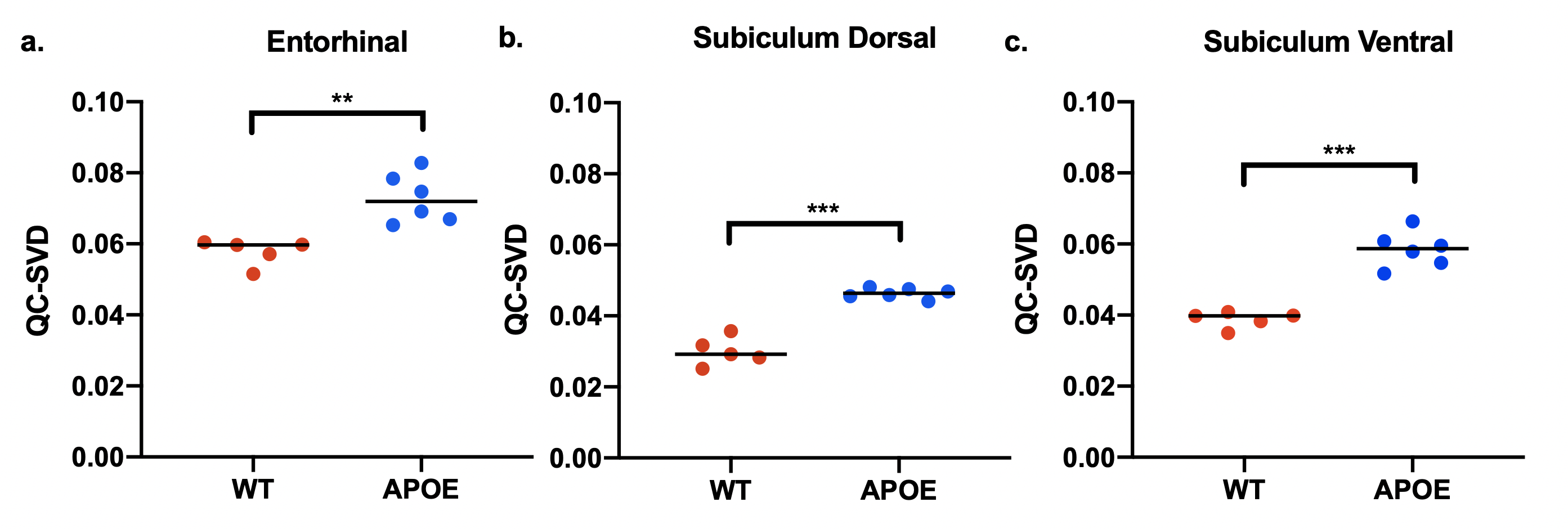


Supplementary Figure 10. Additional regions associated with tri-synaptic circuit function. The subiculum and Entorhinal cortex were both highly impacted in the APOE4 rat model. Statistically analyses were performed with ANOVA.
