# Supplementary material for: Neurovascular imaging with QUTE-CE MRI in APOE4 rats reveals early vascular abnormalities
Source: PLoS One. 2021 Aug 27;16(8):e0256749. doi: 10.1371/journal.pone.0256749 (PMC8396782; doi:10.1371/journal.pone.0256749)
Supplement: S11 Fig — In order to confirm that blood-brain barrier leakage was not a confound in our experiment, we later assessed available APOE4 rats (n = 5) that were slightly older (16 months) and on a high fat diet- both of which would exacerbate BBB leakage if present. (a) The average QC-SVD is displayed when sampling the entire rat brain over time. (b-g) QC-SVD over time for various regions associated with the hippocampus. At 16 months of age. no visible BBB leakage was detected, and this was confirmed (p>0.05) with a linear regression model, testing for an increase in slope over the first 5 scans at the whole brain level. (DOCX) [file pone.0256749.s011.docx]

**
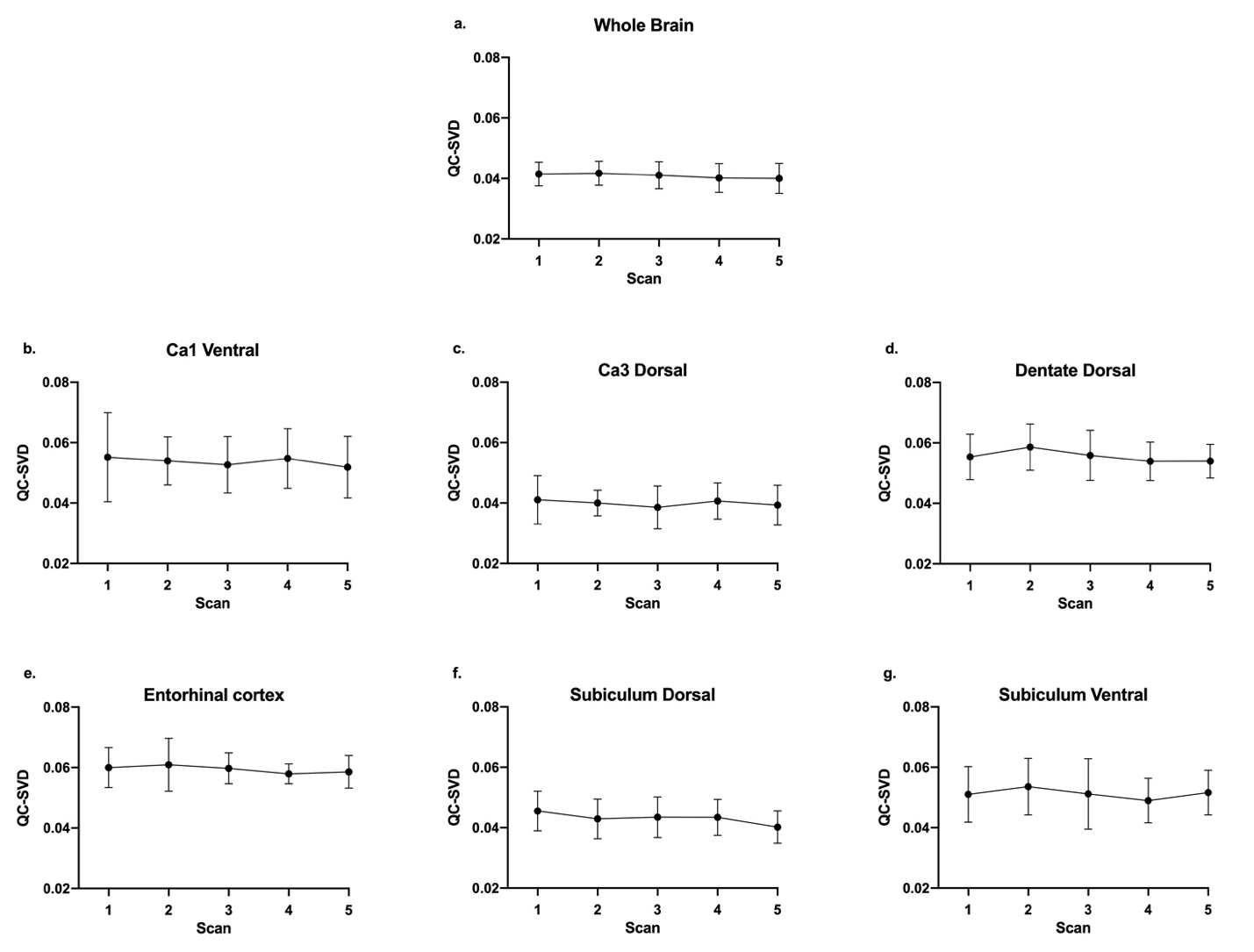
**

Supplementary Figure 11. BBB leakage assessment. In order to confirm that blood-brain barrier leakage was not a confound in our experiment, we later assessed available APOE4 rats (n=5) that were slightly older (16 months) and on a high fat diet- both of which would exacerbate BBB leakage if present. (a) The average QC-SVD is displayed when sampling the entire rat brain over time. (b-g) QC-SVD over time for various regions associated with the hippocampus. At 16 months of age. no visible BBB leakage was detected, and this was confirmed (p>0.05) with a linear regression model, testing for an increase in slope over the first 5 scans at the whole brain level.
