# Supplementary material for: Neurovascular imaging with QUTE-CE MRI in APOE4 rats reveals early vascular abnormalities
Source: PLoS One. 2021 Aug 27;16(8):e0256749. doi: 10.1371/journal.pone.0256749 (PMC8396782; doi:10.1371/journal.pone.0256749)
Supplement: S1 Table — Linear regression analysis was performed to check if any region had a statistically significant increase in slope over 5 scans, which is an indicator for BBB leakage. Across 5 APOE animals, only 1 region (gigantocellular reticular nucleus, 1.07% brain volume) of 1 animal indicated a statistically significant increase in signal over time. (DOCX) [file pone.0256749.s012.docx]

|  | **APOE-hfd** | |
| --- | --- | --- |
|  | # Regions | Brain Volume |
| Patient 1 | 0 | 0.00% |
| Patient 2 | 0 | 0.00% |
| Patient 3 | 0 | 0.00% |
| Patient 4 | 1 | 1.07% |
| Patient 5 | 0 | 0.00% |

Supplementary Table 1. HFD BBB leakage assessment summary. Linear regression analysis was performed to check if any region had a statistically significant increase in slope over 5 scans, which is an indicator for BBB leakage. Across 5 APOE animals, only 1 region (gigantocellular reticular nucleus, 1.07% brain volume) of 1 animal indicated a statistically significant increase in signal over time.
